# Cephalic Vein Cutdown Is Superior to Subclavian Puncture as Venous Access for Patients with Cardiac Implantable Devices after Long-Term Follow-Up

**DOI:** 10.3390/jcm13041044

**Published:** 2024-02-12

**Authors:** Dario Knorr, Dirk Bandorski, Harilaos Bogossian, Konstantinos Iliodromitis, Fabian Schiedat, Zana Karosiene, Dejan Mijic, Bernd Lemke, Melchior Seyfarth, Sabrina Voß, Stephanie Knippschild, Assem Aweimer, Markus Zarse, Axel Kloppe, Spiridon Botsios

**Affiliations:** 1 Department of Cardiology, University Witten/Herdecke, 58455 Witten, Germany; dario.knorr@googlemail.com (D.K.); konstantinos.iliodromitis@gmail.com (K.I.); melchiior.seyfarth@helios-gesundheit.de (M.S.); markus.zarse@klinikum-luedenscheid.de (M.Z.); botsios@arcor.de (S.B.); 2 Faculty of Medicine, Semmelweis University, 1085 Budapest, Hungary; dirk.bandorski@t-online.de; 3 Cardiology and Rhythmology, Ev. Krankenhaus Hagen, 58135 Hagen, Germany; 4 Department of Cardiology and Angiology, Bergmannsheil University Hospitals, Ruhr University of Bochum, 44789 Bochum, Germany; fschiedat@aol.com (F.S.); assem.aweimer@rub.de (A.A.); a.kloppe@marienhospital.eu (A.K.); 5 Department of Cardiology and Angiology, Marienhospital Gelsenkirchen, 45886 Gelsenkirchen, Germany; 6 Department of Cardiology, Elektrophysiology and Angiology, Klinikum Lüdenscheid, 58515 Luedenscheid, Germany; zana.karosiene@klinikum-luedenscheid.de (Z.K.); dejan.mijic@klinikum-luedenscheid.de (D.M.); bernd.lemke@rub.de (B.L.); 7 Department of Cardiology, Helios Klinikum Wuppertal, 42283 Wuppertal, Germany; 8 Faculty of Health, Institute for Medical Biometry and Epidemiology, Witten/Herdecke University, 58455 Witten, Germanystephanie.knippschild@uni-wh.de (S.K.)

**Keywords:** phlebography, CIED, cephalic vein cutdown, subclavian puncture, vein occlusion

## Abstract

Background: Cephalic vein cutdown (CVC) and subclavian vein puncture (SVP) are the most commonly used access sites for transvenous lead placement of cardiac implantable electronic devices (CIEDs). Limited knowledge exists about the long-term patency of the vascular lumen housing the leads. Methods: Among the 2703 patients who underwent CIED procedures between 2005 and 2013, we evaluated the phlebographies of 162 patients scheduled for an elective CIED replacement (median of 6.4 years after the first operation). The phlebographies were divided into four stenosis types: Type I = 0%, Type II = 1–69%, Type III = 70–99%, and Type IV = occlusion. Due to the fact that no standardized stenosis categorization exists, experienced physicians in consensus with the involved team made the applied distribution. The primary endpoint was the occurrence of stenosis Type III or IV in the CVC group and in the SVP group. Results: In total, 162 patients with venography were enrolled in this study. The prevalence of high-degree stenosis was significantly lower in the CVC group (7/89, 7.8%) than in the SVP group (15/73, 20.5%, *p* = 0.023). In the CVC group, venographies showed a lower median stenosis (33%) than in the SVP group (median 42%). Conclusions: The present study showed that the long-term patency of the subclavian vein is higher after CVC than after SVP for venous access in patients with CIED.

## 1. Introduction

Cardiac implantable electronic devices (CIEDs) require transvenous access for lead placement in the chambers of the heart. Subsequent lead placement or extraction may be required due to lead damage or dysfunction, upgrades to the biventricular pacing system, or CIED-related endocarditis during the patient’s lifetime. Adequate luminal patency of the ipsilateral subclavian vein of the initial CIED implantation is of paramount importance for a successful and uneventful procedure in subsequent procedures. The two most commonly used venous access sites for CIED lead placement are the cephalic and subclavian veins [1]. In recent years, axillary puncture seems to have become a possible alternative. Although direct subclavian vein puncture may result in reduced lead longevity [2,3] and has been shown to be associated with higher rates of pneumothorax and bleeding, it requires less time and surgical skills and is always available [4,5]. Despite most physicians being very familiar with the Seldinger technique, the risk of injury to adjacent anatomic structures (like muscles, arteries, or the brachial plexus), inadvertent arterial puncture, pneumothorax, or hemothorax during venous puncture of the subclavian vein is still an often-seen complication. In the literature, the risk of pneumothorax after subclavian vein access is described as being 7.8-fold increased compared to the cephalic vein cutdown access [6].

These risks could be avoided by using an alternative access. The axillary vein access offers more safety due the extra-thoracic puncture. Liu et al. described during the perioperative period and follow-ups a significantly decreased incidence of complications after axillary vein access versus the subclavian vein approach [1.6% (2/125) versus 8.2% (10/122)] [7]. Retrospective non-randomized data also described a lower rate of lead failure after implantation via the axillary vein route. Thereby, lead failure occurred in 1.2% of patients with axillary vein puncture, compared to 5.6% of patients with subclavian vein puncture, in a mean follow-up of 73.6 ± 33.1 months [3]. However, the long-term randomized, prospective follow-up data are still limited, especially regarding the sharper angel in the rout of the leads. The cephalic vein approach allows direct vision during venous insertion of the leads and abandons, by this way, most of the complications. Additionally, access through the cephalic vein, on the other hand, results in better CIED lead longevity and fewer perioperative complications but takes more time, requires more surgical preparation, and is less suitable [1]. These challenges are responsible for the decreased success rates in the cephalic vein approach. Chen NY et al. described in a retrospective cohort study the lowest success rate for the cephalic vein cutdown (subclavian vein 96.8%, axillary vein 97.6%, cephalic vein cutdown 78.2%) [3].

Seldom are signs of venous occlusion visible before the procedure. In these cases, pre-operative imaging may be helpful in strategic planning of alternative venous access (e.g., (venography or CT scan). Rarely is the alternative access via transfemoral or epicardial approach necessary. In all the different approaches, ultrasound guidance (especially for axillary vein puncture) could be a helpful technique for increasing safety and reducing complications [8].

Currently, limited data exist regarding the luminal patency of the subclavian vein housing the CIED leads. These are important information for operators and their patients, who may need lead extraction in the future. An alternative to lead extraction could be capping and abandoning unnecessary leads. However, this strategy is associated with a higher infection rate over a period of years [9]. Data from the European lead extraction registry additionally described the occlusion or the thrombosis of the superior venous access as an independent predictor for cardiac tamponade and major vascular complications [10]. The aim of the present retrospective study was to examine the long-term occurrence and degree of luminal distortion of the subclavian vein after using a CIED lead.

## 2. Materials and Methods

### 2.1. Study Population

The aim of the present study was to analyze the effects of venous access on the patency of the ipsilateral venous system in long-term follow-up. In particular, the study aimed to analyze of the grade of stenosis in the subclavian vein based on the applied access site for the initial CIED lead placement (cephalic vein cutdown versus puncture of the subclavian vein). With this scope, the cardiology database of the Luedenscheid Hospital (Germany) was screened retrospectively.

All patients who underwent a second CIED implantation or device upgrade between 2005 and 2013 were identified. If a phlebography was performed before the operation and the operation protocol of the first implantation was present, the patient was included for the final analysis. Klinikum Luedenscheid is a high-volume center for treatment of diverse arrhythmia disorders and is especially associated with high experience for device treatment. To prevent unexpected complications during the planned procedure, there was a standard protocol to perform phlebographies whenever possible and in absence of obvious contraindications. The median time between first implantation and phlebography was 6.4 years.

**Figure 1:**
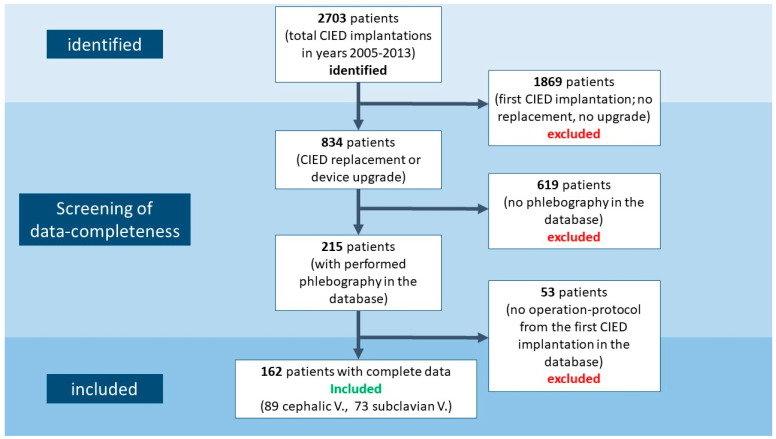
Study flowchart.

All patients provided informed consent prior to the phlebography and the CIED operation for analysis of their data. Out of 2703 patients who were operated on for CIED implantation (from 2005–2013), 1896 patients were excluded, as the performed procedure was the primary device implantation. Among the remaining 834 patients who underwent CIED replacement or device upgrade, phlebography data were available for 215 patients. The operation protocol of the first implantation was available for 162 patients (Figure 1).

The study conforms to the guiding principles of the Declaration of Helsinki. The study protocol was approved by the local ethics committee. Due to the retrospective character of the study, the local ethic committee waived patient informed consent, especially since the study is of a retrospective nature without any consequences relating to patient management, and without active follow-up.

### 2.2. Phlebography

Prior to scheduled CIED generator change, upgrade, or lead revision, phlebographies were performed routinely in our hospital. A standard 1.3-gauge venous cannula was placed on the ipsilateral upper extremity. Complete cineangiography of the axillary vein, the subclavian vein, and the brachiocephalic trunk was obtained after the forced injection of 20 mL of contrast medium. Before the injection, a tourniquet was place at the upper arm. Immediate after the injection, the tourniquet was released and an additional flush with 20 mL saline solution followed.

**Figure 2:**
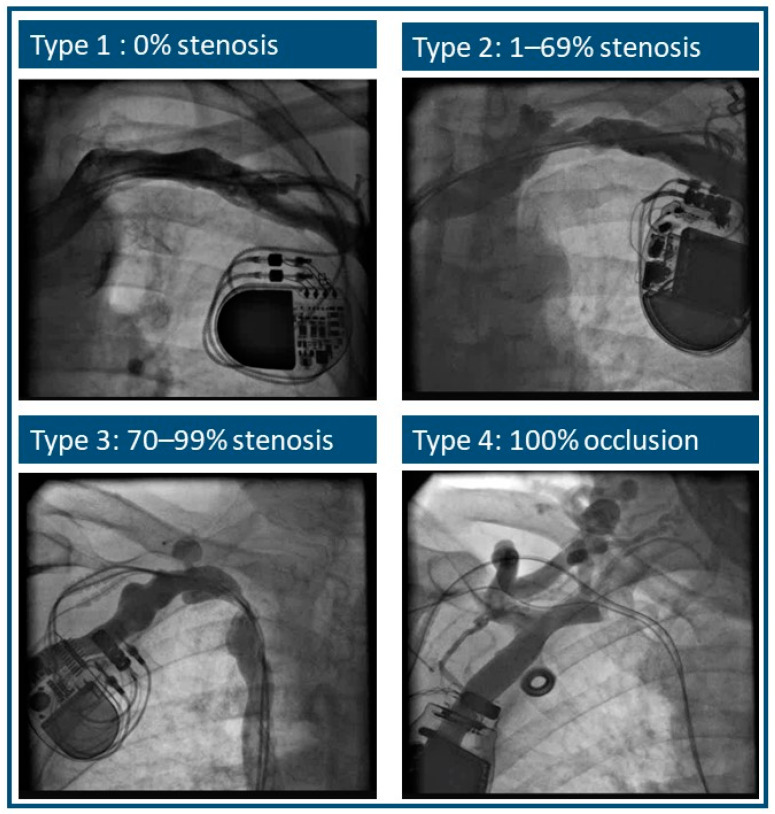
Grade of stenosis (Type 1: no stenosis; Type 2: 1–69% stenosis; Type 3: 70–99% stenosis; Type 4: total occlusion).

Due to the fact that no standardized stenosis categorization exists, experienced physicians in consensus with the involved team made the used distribution. Vein patency was assessed, and vein distortion was graded into four types: Type 1 represented patent veins without obstruction (0%); Type 2 included cases with subclavian vein stenosis ranging between 1% and 69%; Type 3 included patients with stenosis severity between 70% and 99%; and Type 4 represented cases with complete occlusion of the vein (Figure 2).

The degree of subclavian vein stenosis was calculated by comparing its narrowest lumen diameter (in the stenosis) with the mean value of the lumen diameter proximal and distal to the stenosis with the following formula:

Q_S_ = 1 − D_S_ ÷ 50% × (D_D_ + D_P_)

where the following are defined: Q_S_: stenosis ratio; D_S_: stenosis diameter; D_D_: distal/pre-stenotic diameter; D_P_: proximal/post-stenotic diameter.

As some phlebographies were performed in external radiology departments or were older than 10 years, only 215 phlebographies were considered appropriate.

### 2.3. Primary Endpoint and Sample Size Calculation

The primary endpoint was the occurrence of a venous occlusion with stenosis Type III or IV. To show an expected difference in venous occlusion (stenosis Type III or IV) of 30% (subclavian) to 10% (cephalic) to a significance level of 5% and a power of 80% with a two-sided Fisher test, at least 69 people per group had to be included in the study.

### 2.4. Statistical Analysis

The descriptive primary analysis was carried out by calculating the frequency and odds ratio of the occurrence of stenosis of grade I or II versus III or IV. Fisher’s exact test was used to determine whether there was a significant difference in the incidence of stenosis of grade III or IV between the two groups. The level of significance for this comparison was 5%.

Additional descriptive analyses were carried out by determining relative and absolute frequencies and using boxplots.

The applied software was R Version 3.4.0 Core Team (2017) [11]. 

## 3. Results

Data from a total of 162 patients scheduled for a second CIED implantation were analyzed. The basic characteristics are shown in Table 1.

Seventy-four percent of the patients were male, and the median age was 73 years (Q1–Q3: 67–79). The patients presented due to a CIED replacement, upgrade, or lead revision from a pacemaker (*n* = 70) or an ICD (*n* = 92).

Based on the operation protocols, patients were categorized into two groups: the cephalic vein cutdown group for the initial CIED implantation included 89 patients, and the subclavian puncture group for the initial CIED implantation included 73 patients. The basic characteristics, medical history, and cardiomyopathic history of all patients are shown in Table 1.

Due to the retrospective nature of the study, the basic characteristics were presented in a descriptive manner. Patients in the cephalic vein group presented with a higher percentage with oral anticoagulation treatment compared to the subclavian vein group (38% versus 30%). In contrast, in the subclavian vein group, more patients were treated with antiplatelets compared to the cephalic vein group: subclavian vein group with ASA (45%) and Clopidogrel (16%) versus cephalic vein group with ASA (31%) and Clopidogrel (13%).

A high-degree stenosis Type (III and IV), which was the primary endpoint of this study, was observed in 8% (7 out of 89) of patients in the cephalic vein cutdown group and 21% (15 out of 73) of patients in the subclavian vein puncture group (Table 2).

The odds ratio was higher and had a value of 3.03 for the risk of developing stenosis Type III or IV in the subclavian vein puncture group. Fisher’s exact test yielded a *p* value of 0.02, which was less than 0.05; therefore, a significant difference in the occurrence of stenosis Type III or IV was found between the two groups.

The evaluated phlebographies revealed a lower stenosis ratio in the cephalic vein cutdown group than in the subclavian vein puncture group (33% [Q1–Q3: 22–36%] vs. 42% [Q1–Q3: 24–47%]). The distribution of the different stenosis types is shown in Table 3.

**Figure 3:**
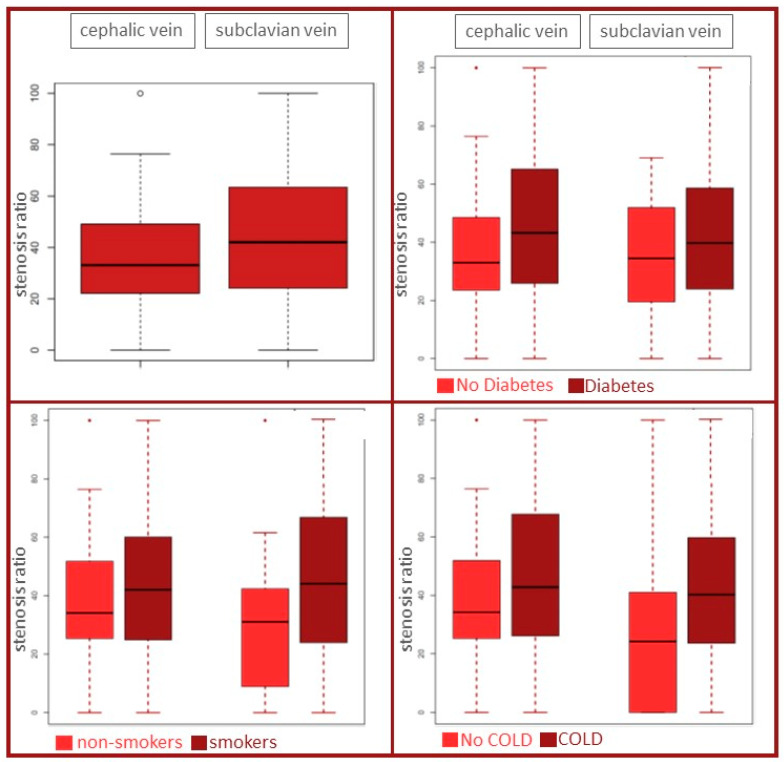
Comparison of the stenosis ratio between the cephalic vein and subclavian puncture groups (**left upper panel**). Influence of diabetes mellitus, smoking, and chronic obstructive lung disease (COLD) on the stenosis ratio (**right upper panel** and **two lower panels**). The symbols (circle and dots) represent outliers with values that differ from most (the middle) of the other values.

**Figure 4:**
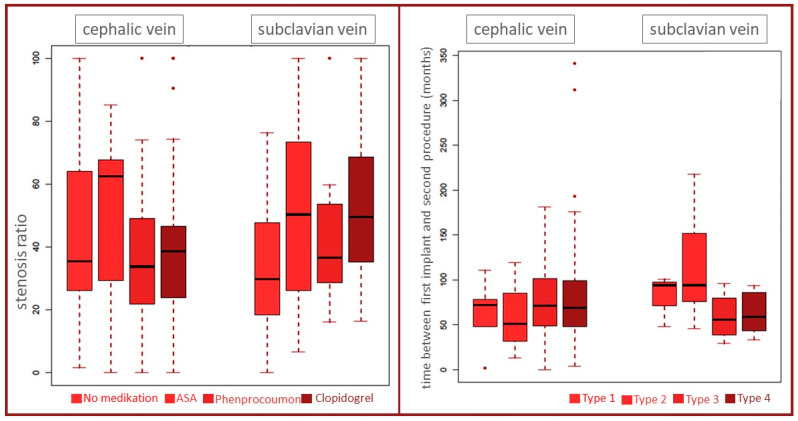
Comparison of the stenosis ratio between the cephalic vein and subclavian puncture groups dependent on medication (**left panel**). Influence of time between first CIED implantation and second procedure on stenosis types (**right panel**). The dots represent outliers with values that differ from most (the middle) of the other values.

There were no relevant differences in comorbidities or in the timely distance between the first CIED implantation and the second operation (Figure 3 and Figure 4). Figure 3 depicts the comparison between diabetes and non-diabetes, and between smokers and non-smokers, as well as between COLD and no COLD in the cephalic vein group and the subclavian vein group, respectively. Figure 4 depicts beside the antiplatelet and anticoagulation therapy, especially the time passed between the index operation and the second procedure, when phlebography was performed (mean in cephalic vein group 74 ± 38 months versus 83 ± 60 months in the subclavian vein group, respectively).

Regarding the parameter “number of pacemaker or ICD leads”, the resulting groups were too small to analyze the impact of different leads on the stenosis ratio. The distribution is presented in Table 4. Stenosis of Type 3 and 4 occurred in patients with one, two, or three leads and independent of the device (occurred in both Pacemakers and ICDs).

## 4. Discussion

The implantation of cardiac electronic devices is a common and increasingly performed procedure in clinical cardiology [4,5,12]. Despite the increasingly aging population, younger patients with underlying cardiac disease may require CIED therapy and often subsequent implantations during their lifetime. Although single device exchange after battery depletion remains a rather uncomplicated procedure, additional lead implantation, lead revision and extraction, or upgrading to the biventricular pacing system may be necessary [13,14]. Both insulation damage and fracture of a CIED lead are serious events with deleterious or even life-threatening effects [2]. Thus, maintaining an easily accessible and patent subclavian vein ipsilateral to the side of initial implantation is of paramount importance. There are three sites for vascular access currently used for permanent transvenous lead pacing: the cephalic, subclavian, and axillary veins. Although the superiority of cephalic and axillary vein access for CIED lead longevity compared to subclavian vein access has been previously demonstrated [3], there is limited knowledge of the degree of subclavian vein stenosis over time among each transvenous route [15].

The association between venous stenosis and the number and diameter of implanted leads has been described previously, as well as the impact of increasing incidence for venous stenosis due to multiple implant procedures [16]. However, the additional value of the preferred access route in the index procedure has not been described before.

Chan et al. demonstrated the importance of the initial choice for intravascular access for lead longevity [3]. In the follow-up of 73.6 ± 33.1 months, the authors identified a 5.6% lead failure rate in those implanted via subclavian puncture in 681 implanted pacemakers versus 1.2% in the axillary puncture group and 2.3% in the cephalic vein cutdown group. Benz et al. showed in a meta-analysis that cephalic vein cutdown is superior to subclavian puncture regarding perioperative complications [15]. In a total of 20 included studies, more than 50,000 leads implanted via cephalic vein or subclavian puncture were analyzed and showed that the incidence of pneumothorax and lead failure was higher in the subclavian puncture group (0.19% vs. 1.3% and 0.5% vs. 1.92%, respectively; both *p* < 0.001). Our study is the first to demonstrate an impact of the initially chosen venous access site in the index device implantation procedure on the future patency of the venous system. After a median period of 6.4 years, an initial access using the cephalic vein cutdown resulted in a significantly more patent subclavian vein. Patients for whom the subclavian route was initially chosen developed a pronounced reduction in their subclavian vein diameter.

Possible explanations for less favorable outcomes (especially lead fracture) after transvenous CIED lead implantation via the subclavian route have been well described. The most important unfavorable outcome is the limited anatomic space between the clavicle and the first rib. This is caused by further relevant compressive forces, stressing the leads (e.g., muscles, ligaments, and bones) [1,17]. The risk of injury of the subclavian vein, the subclavian muscle, and the adjacent ligaments increases with subclavian puncture, especially when performed more medially by targeting the groove between the clavicle and first rib [18]. Chronic irritation of the area promotes the development of increased fibrotic tissue and ossification. The aforementioned processes may “strangulate” the shaft of the lead, subdue it to higher shear forces and damage it prematurely [19,20]. Damage of the CIED lead inserted via the subclavian vein has also been suggested in cadavers as a result of soft tissue entrapment, causing repeated flexion of the leads during movements [2]. These findings are in accordance with our angiographic findings. More severe lumen reduction was observed in patients with initial subclavian vein puncture. This may be a result of increased intraluminal endothelial proliferation and/or thrombus formation and extraluminal compression from tissue growth. However, the mechanism for development of vein stenosis or occlusion during mid/long-term follow-up with the subclavian access is not absolutely clear. Possibly, some intimal injury of the vein can be additionally be discussed. On the other hand, the fact that a subclavian access had to be used may translate to smaller veins, which per se will be more prone to the development of stenosis or even occlusion in the long term. In this regard, the subclavian vein was used significantly more frequently in female patients, who probably have smaller veins, and more prone to stenosis during follow-up. This in combination with the described stress, injury, fibrosis, and ossification may complete the pathophysiology of the presented results.

Despite the increased risk of a less favorable long-term outcome after CIED lead implantation via the subclavian vein, this approach is frequently utilized in current practice [4,5]. There are limited data regarding the impact of common comorbidities on the severity of subclavian vein stenosis in patients with transvenous CIED. Comorbidities such as diabetes, arterial hypertension, COPD, and tobacco consumption failed to show an increased severity of subclavian vein stenosis in our study. Additionally, the presence of ongoing medication with antiplatelet and anticoagulant agents does not seem to result in lesser degrees of subclavian vein stenosis in either group of patients. However, as the primary focus was not on comorbidities, the study may be underpowered to answer these questions. Nevertheless, we see a tendency of higher amount with oral anticoagulation treatment in the cephalic vein group compared to the subclavian vein group (38% versus 30%). Regarding the antiplatelet medication, there was a tendency for more ASA and Clopidogrel treatment in the subclavian vein group compared to the cephalic vein group (ASA: 45% versus 31%; Clopidogrel: 16% versus 13%).

Data from lead extraction registries have demonstrated that long indwelling CIED leads are associated with an increased risk of lead damage and complicated extraction [21,22]. In the present study, the median time between the first transvenous CIED implantation and subclavian vein angiography was 6.4 years. The time frame alone does not seem to be a significant factor for the grade of subclavian vein stenosis, as demonstrated in the subgroup analysis. In contrast, the initial implantation technique is important for the future occurrence of a higher stenosis rate. Therefore, it is important to prevent additional thrombotic alterations in the subclavian region by avoiding the subclavian vein puncture technique during the first procedure. In the future, further improvement of leadless technologies could offer the solution to avoid venous complications caused by lead placement. The development from a single- to dual-chamber pacemaker is very promising [23,24]. However, regarding ICD therapy, hope centers on subcutaneous and wearable technologies [25,26].

Finally, although cephalic vein cutdown should be preferable for CIED lead introduction [27,28] and may even be suitable for housing all three leads of biventricular pacing systems [29], anatomical variances may make the cephalic vein an inappropriate access site. Our findings are in accordance with the recently published guidelines on cardiac pacing and resynchronization therapy from the European Society of Cardiology, which proposes opting not to use subclavian vein access [27,30,31,32]. A possible alternative is the direct puncture of the axillary vein instead of the subclavian vein, as discussed in the guidelines. Unfortunately, this access was not often used in the years of our data acquisition; therefore, we cannot make a statement regarding the effects of axillary access on the venous system over the years included in our data. The present study has an additional value and generates further hypothesis for future research. However, due to the retrospective nature of the study, it is difficult to phrase hard recommendations. Further prospective studies are necessary to support guideline recommendations in the future.

## 5. Limitations

This is a single-center observational, retrospective, nonrandomized study. The observed heterogeneity between the groups may have led us to under- or overestimate the current findings. Due to the multiple different patient characteristics, a much higher number of patients would be necessary to confirm causality. Furthermore, the accurate analysis of the initial CIED implantation as well as the availability of phlebographies many years after index implantation limited the number of eligible patients. Furthermore, there still could be a bias regarding the selection of the patients in whom phlebography was performed. Although phlebography was a standard procedure before planned replacements, some patients may have contraindications, or due to a very short hospital stay, phlebography may be skipped. Finally, the study did not have the power to analyze further comorbidities, and additionally, the power limits the desired extensive statistical workup. Therefore, these data are presented only in a descriptive manner.

## 6. Conclusions

CIED implantation is a daily applied therapy for the treatment of bradycardias by pacemakers and for the prevention of sudden cardiac death by ICDs. Although both therapies have, over the years, become commonly used operations, there is a lack of a standard approach regarding the venous access of the implanted leads. Operators decide according to their individual preference about access via subclavian vein puncture, axillary vein puncture, or cephalic vein cutdown.

Besides the acute complications and success rates, physicians should also consider the potential long-term complications and outcomes. For the choice of the best or a standardized strategy, sufficient prospective, randomized data are unsatisfactory.

The presented data offer additional evidence for the effects of the used access route on the long-term outcome of the venous alterations over a period of years.

Venous access using cephalic vein cutdown for the implantation of a CIED was associated with a statistically significantly higher patency of the ipsilateral subclavian vein compared to subclavian puncture as the vein access method after a median period of 6.4 years.

## Figures and Tables

**Table 1:** Basic characteristics, ECG, medication, and cardiomyopathies.

	Cephalic Vein	Subclavian Vein
	*n* = 89	*n* = 73
Basic characteristics		
female	16 (18%)	26 (36%)
age	73 ± 7	70 ± 7
smoker	23 (26%)	18 (25%)
adipositas	40 (45%)	34 (47%)
Diabetes	31 (35%)	29 (40%)
hypertension	81 (91%)	66 (90%)
HLP	64 (72%)	48 (66%)
CAD	52 (58%)	45 (62%)
renal failure	29 (33%)	26 (36%)
COLD	18 (20%)	24 (33%)
ECG		
SR	64 (72%)	49 (67%)
AF	25 (28%)	24 (33%)
AVB	31 (26%)	21 (29%)
Medication		
ASA	31 (35%)	33 (45%)
Phenprocoumon	34 (38%)	22 (30%)
Clopidogrel	12 (13%)	12 (16%)
Cardiomyopathy		
ICM	26 (29%)	16 (22%)
DCM	13 (15%)	15 (21%)
HCM	3 (3%)	2 (3%)
myocarditis	1 (1%)	0

AF: atrial fibrillation; ASA: acetyl salicylic acid; AVB: atrioventricular block; CAD: coronary artery disease; COLD: chronic obstructive lung disease; DCM: dilated cardiomyopathy; HCM: hypertrophic cardiomyopathy; HLP: hyperlipidemia; ICM: ischemic cardiomyopathy; SR: sinus rhythm.

**Table 2:** Frequencies of stenosis Type 1 or 2 and stenosis Type 3 or 4 in the two groups.

Types of Stenosis	Cephalic Vein	Subclavian Vein	Total
1 and 2	82 (92%)	58 (79%)	140
3 and 4	7 (8%)	15 (21%)	22
total	89	73	162

**Table 3:** Mean stenosis ratio and types of stenosis.

	Cephalic Vein (*n* = 89)	Subclavian Vein (*n* = 73)
Mean stenosis ration		
	36 ± 13%	46 ± 17%
Types of stenosis		
1 (stenosis 0%)	6 (6.7%)	3 (4.1%)
2 (stenosis 1–69%)	76 (85.4%)	55 (75.3%
3 (stenosis 70–99%)	3 (3.4%)	9 (12.3%)
4 (occlusion)	4 (4.5%)	6 (8.2%)

**Table 4:** Distribution of stenosis types based on the numbers of leads (ICD: implantable cardioverter defibrillator).

Stenosis Type	Number of Leads					
	Pacemaker			ICD		
	1 Lead	2 Leads	3 Leads	1 Lead	2 Leads	3 Leads
	*n* = 9	*n* = 51	*n* = 10	*n* = 28	*n* = 21	*n* = 43
Type 1	0	3	0	2	0	4
Type 2	7	42	7	23	18	34
Type 3	1	4	2	2	2	1
Type 4	1	2	1	1	1	4

## Data Availability

No new data were created or analyzed in this study. Data sharing is not applicable to this article.
